# A selected population study reveals the biochemical mechanism of intramuscular fat deposition in chicken meat

**DOI:** 10.1186/s40104-022-00705-3

**Published:** 2022-05-12

**Authors:** Huanxian Cui, Lu Liu, Xiaojing Liu, Yongli Wang, Na Luo, Xiaodong Tan, Yuting Zhu, Ranran Liu, Guiping Zhao, Jie Wen

**Affiliations:** 1 State Key Laboratory of Animal Nutrition; Institute of Animal Science, Chinese Academy of Agricultural Sciences, Beijing, 100193 China; 2 College of Animal Science and Technology, College of Veterinary Medicine of Zhejiang A&F University, Hangzhou, 311300 China

**Keywords:** Biochemical mechanism, Chicken, Fatty acid composition, Intramuscular fat

## Abstract

**Background:**

Increasing intramuscular fat (IMF) is an important strategy to improve meat quality, but the regulation mechanism of IMF deposition needs to be systematically clarified.

**Results:**

A total of 520 chickens from a selected line with improved IMF content and a control line were used to investigate the biochemical mechanism of IMF deposition in chickens. The results showed that the increased IMF would improve the flavor and tenderness quality of chicken meat. IMF content was mainly determined both by measuring triglyceride (TG) and phospholipid (PLIP) in muscle tissue, but only TG content was found to be decisive for IMF deposition. Furthermore, the increase in major fatty acid (FA) components in IMF is mainly derived from TGs (including C16:0, C16:1, C18:1n9c, and C18:2n6c*,* etc.*)*, and the inhibition of certain very-long-chain FAs would help to IMF/TG deposition.

**Conclusions:**

Our study elucidated the underlying biochemical mechanism of IMF deposition in chicken: Prevalent accumulation of long-chain FAs and inhibitions of medium-chain FAs and very long chain FA would jointly result in the increase of TGs with the FA biosynthesis and cellular uptake ways. Our findings will guide the production of high-quality chicken meat.

**Supplementary Information:**

The online version contains supplementary material available at 10.1186/s40104-022-00705-3.

## Background

As a result of its deliciousness, tenderness, and juicy quality, chicken is the second most-consumed meat worldwide by humans. With the use of continuous growth breeding and high-density feeding to produce more meat, the meat quality deteriorated due to the relatively higher water and lower fat content in the meat. Currently, the challenges of improving meat quality are the focus of intensive scientific research [1, 2]. Intramuscular fat (IMF), a mixture of various lipids (including triglyceride (TG), phospholipid (PLIP), total cholesterols (TCHO), etc.) in muscle tissue, which has an important role in determining the flavor, tenderness, and water holding capacity of meat [3–6]. For example, Kobe beef is known for its high meat quality grade which is determined by its high beef marbling score (BMS).

Fatty acids (FAs), as a basic component of cells by forming TG and PLIP, besides being important structural components for cells, play an important regulatory role in animal biological processes [7–9], including the energy storage, information transfer and regulation of metabolism. The sources of FAs in animal tissues include cellular uptake and de novo lipogenesis (DNL). FAs for cellular uptake are derived from blood lipids through lipoprotein lipase (LPL)*-*mediated hydrolysis [10, 11]. However, carbon-16 FA (palmitic acid) is synthesized through the intracellular DNL pathway in a reaction catalyzed by FA synthase (FASN) using acetyl coenzyme A and malonyl coenzyme A as substrates, and then the carbon chain elongation is regulated by multiple proteins [12].

Due to the absence of the marbling typically found in chicken meat, the deposition of IMF in chicken cannot be directly identified anatomically and is thus difficult to accurately determine. These factors have greatly restricted the characterization of IMF deposition in chicken. As it is well known, DNL mainly occurs in the liver in poultry [13], and the necessary FAs are mainly provided by cellular uptake in other tissues. Moreover, in general, the muscle tissue mainly includes myocyte, adipocyte and connective tissue cells. Unlike abdominal fat, the formation of IMF is more complicated due to the interference of myocytes, thus more research is required for the elucidation of the underlying biochemical mechanism of IMF deposition in chickens.

A useful experimental model for elucidating the biochemical mechanism of IMF formation in chickens is advantageous [14, 15]. Our research group had artificially bred a selected population of Jingxing yellow-feather chickens (JXY, which is a synthetic line derived from a local Chinese chicken) with the IMF content in breast tissue as the main selection trait [15]. In this study, we used this selected line at generation 16 and the corresponding control line of JXY chickens to systematically investigate the biochemical mechanism of IMF deposition. It is anticipated that our research will not only enrich the theory on IMF formation, but will also provide guidance for future production of high-quality broilers.

## Materials and methods

### Ethics statement

This study was conducted in accordance with the Guidelines for Use of Experimental Animals established by the Ministry of Science and Technology (Beijing, China). All experimental protocols were approved by the Science Research Department (in charge of animal welfare issues) of the Institute of Animal Sciences, Chinese Academy of Agricultural Sciences (CAAS; Beijing, China) (No. IAS2019–21).

### Animals and sample collection

A total of 520 female JXY chickens (Selected line: *n* = 256; Control line: *n* = 264) obtained from the Institute of Animal Science, CAAS (Beijing, China) were used in this study. The two lines of JXY chickens originated from the same base population of JXY100, the selected line was selected for increased IMF with IMF in breast muscle tissue as the main selection trait, and the control line was randomly bred as previously described [15]. All birds were raised in three-stair step cages (one bird per cage) under the same nutritional condition, and euthanized under carbon dioxide anesthesia by severing the carotid artery at the 98 days of age. Then, the breast muscle tissue was dissected and stored at − 80 °C for subsequent analysis.

### IMF, TG, PLIP and TCHO measurement

To improve the accuracy, the visible fat particles and membranes were removed from the surface of the breast muscle tissue samples of the 520 JXY chickens. After the entire tissue samples were minced and blended, the IMF content (%) was determined by extraction with petroleum ether in a Soxhlet apparatus [16] and expressed as the percentage of the dry weight of breast muscle tissue. A 2.0-g sample of each breast muscle tissue (wet weight) was homogenized and extracted by the method of Folch et al. [17], then TG, PLIP, and TCHO contents were measured using commercially available kits (Beijing Deliman Biochemical Technology Co. Ltd., Beijing, China).

### TG and PLIP separation

Samples of 6.0 g of breast muscle tissue from each of the 8 JXY chickens in the groups with high- or low-IMF content were used for the isolation of TG and PLIP. First, total lipids were extracted by a reported method [17]. Subsequently, TG and PLIP were obtained in turn as previously described [18]. Briefly: a 0.5-mL aliquot of the lipid dissolved in 1 mL of chloroform was placed into an activated aminopropyl silica column, and 2 mL of chloroform-isopropanol solution (volume ratio, 2:1) was added to the column to obtain neutral lipids (mainly TG). Then, 3 mL of 2% acetic acid-diethyl ether (mass to mass ratio) was added to the column to elute the free FA. Finally, 3 mL of methanol was added to elute PLIP. After blowing nitrogen, the obtained TG and PLIP were used for the determination of the FA composition.

### FA composition analysis

A 5.0-g breast muscle tissue sample of each of the 520 JXY chickens, or the separated TG and PLIP were freeze-dried and ground for extraction and methylation of FAs. Then, the FA composition was determined by gas chromatography (GC) according to a method previously reported [19] using an HP6890 gas chromatograph (Agilent Technologies, Santa Clara, CA, USA), and each of FAs was expressed as the percentage of the total fatty acids in breast muscle tissue.

### Meat quality index determination

The meat color, tenderness, and volatile substance of the breast muscle tissue samples of each of the 520 JXY chickens, were determined by a previously described method [15, 19, 20]. Additionally, the flavor quality of the breast muscle tissue samples from each of the 3 birds of the selected line and control line was determined using the electronic nose technology according to a previously described method [21].

### Cells separation and treatment

The different-type primary cells in the pectoralis major muscle of 7-day-old chickens were isolated to obtain the muscle satellite cells and mature adipocyte, using a method based in part on a previous report [22]. Additionally, the mature adipocyte layer was seeded into a 25-cm^2^ cell culture flask containing complete medium. The flask was incubated inverted for 6 d enabling the adipocyte to attach to the upper surface and dedifferentiate, and subsequently re-inverted for another 6 d to obtain preadipocytes [23, 24]. Two types of cells were incubated in a humidified atmosphere of 5% CO_2_ at 37 °C, and the passage 2 cells were seeded into 6-well plates. Two d after reaching 100% confluence, all cells were harvested, and the analysis of TG, PLIP and TCHO contents was performed with the same number of cells.

### RNA sequencing and analysis

Our previous RNA sequencing data (Accession number CRA001908; http://bigd.big.ac.cn/gsa.) of pectoralis from each 8 individuals in the groups with high- or low-IMF content from 520 JXY chickens as previously reported [25] was used in this study. Gene expression levels were determined using the RPKM method. Differentially expressed genes (DEGs) between the two lines were analyzed using the edgeR in the R package and screened by the following criteria: |log_2_ FC| ≥ 0.58, with *Padj* < 0.05.

### Statistical analyses

The significance of the differences between groups was tested by the Student *t*-test using the SPSS Version 22.0 (IBM Corp., Armonk, NY, USA). Confidence limits were set at 95%, and *P* < 0.05 (*) or *P* < 0.01 (**) were considered statistically significant. Data are presented as the mean ± standard error of mean (SEM). The principal component analysis (PCA) and Spearman correlation analysis were performed in R statistical software (version 3.6.1).

## Results

### Increase of IMF content improves the quality of chicken meat

**Fig. 1:**
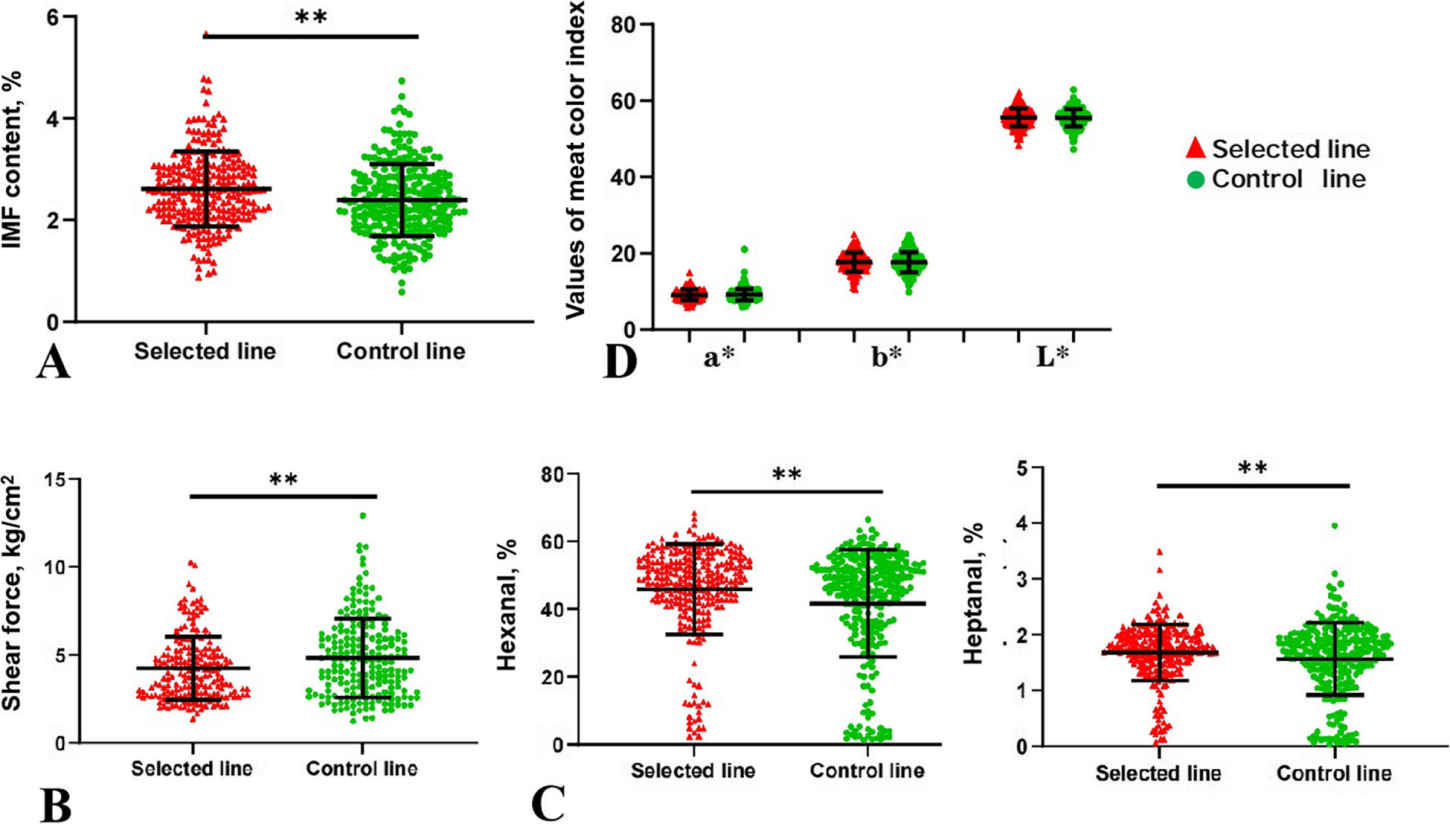
Comparison of IMF content and meat quality between the selected line and the control line. **A** IMF content; **(B)** Shear force; (**C)** Contents of hexanal and heptanal; (**D)** Meat color. Data are expressed as the mean ± standard error of mean (SEM); Selected line: *n* = 256; Control line: *n* = 264

As expected, the results of the analysis of the 520 female JXY chickens from the selected line (*n* = 256) at generation 16 and control line (*n* = 264) showed that the IMF content was more prominently increased in the selected line than that in the control line (*P* < 0.01) (Fig. 1A). In addition, evaluation of the impact of the increased IMF content on the quality of breast meat revealed that the shear force of the breast meat of the selected line was significantly lower than that of the control line (Fig. 1B). Similarly, the contents of two important volatile substances, namely hexanal and heptanal, in the breast meat of the selected line were significantly higher (*P* < 0.01, *P* < 0.05, respectively) than those of the control line (Fig. 1C). However, the meat color of the breast meat was not significantly different between the two lines (Fig. 1D).

### TG controls the IMF content in chicken meat

**Fig. 2:**
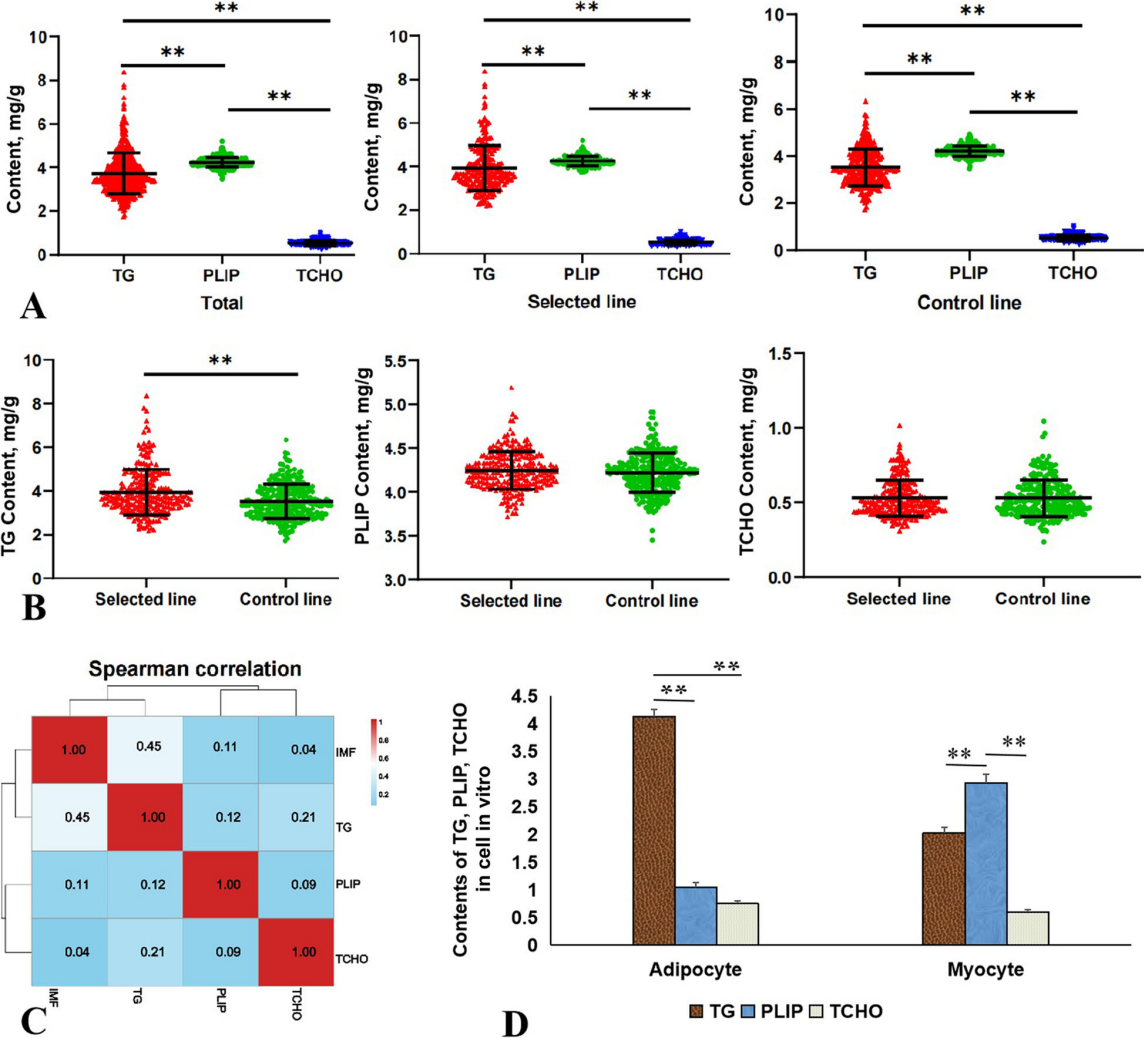
Identification of the decisive ingredients of IMF in chicken meat. **A** Contents of TG, PLIP and TCHO in the selected line and the control line, respectively. The tissue sample (**B).** Comparison of TG, PLIP and TCHO contents between the selected line and the control line. **C** Correlation analysis between IMF, TG, PLIP and TCHO. IMF content had a higher positive correlation with the TG content (*P* < 0.01), and a relatively weak positive correlation with the PLIP content (*P* < 0.05). **D** Contents of TG (mmol/g), PLIP (mmol/L) and TCHO (mmol/gprot) in myocytes or adipocytes derived from the pectoral muscle tissue with the same cell number. Data are expressed as the mean ± standard error of mean (SEM); *n* = 520 individual (in vivo) or 3 (in vitro)

The results on the contents of the main IMF components (TG, PLIP and TCHO) showed that PLIP content was the highest in IMF, and the contents of PLIP and TG were dominant relative to cholesterol that of TCHO (Fig. 2A). Another perspective, the contents of the main IMF components (TG, PLIP and TCHO) between the selected line and the control line revealed, as shown in Fig. 2B, that the contents of PLIP and TG were significantly higher in the breast muscle tissue of the selected line those in the control line (*P* < 0.01, *P* < 0.05, respectively), but the increase of the TG content was higher than that of PLIP. However, the TCHO content was not significantly different between the two lines (*P* > 0.05). Furthermore, Spearman’s correlation analysis showed that the IMF content had a higher positive correlation with the TG content (r = 0.45, *P* < 0.01), and a relatively weak positive correlation with the PLIP content (r = 0.11, *P* < 0.05), but not with the TCHO content (Fig. 2C and Additional file 1: Table S1). In addition, the determination of the contents of TG, PLIP and TCHO in myocytes and adipocytes from the pectoral muscle tissue revealed that in adipocytes the TG content was higher (*P* < 0.01, *P* < 0.01) than those of PLIP and TCHO, while in myocytes the PLIP content was higher (*P* < 0.01, *P* < 0.01) than the TG and TCHO contents (Fig. 2D).

### Change of FA composition determines IMF deposition

**Fig. 3:**
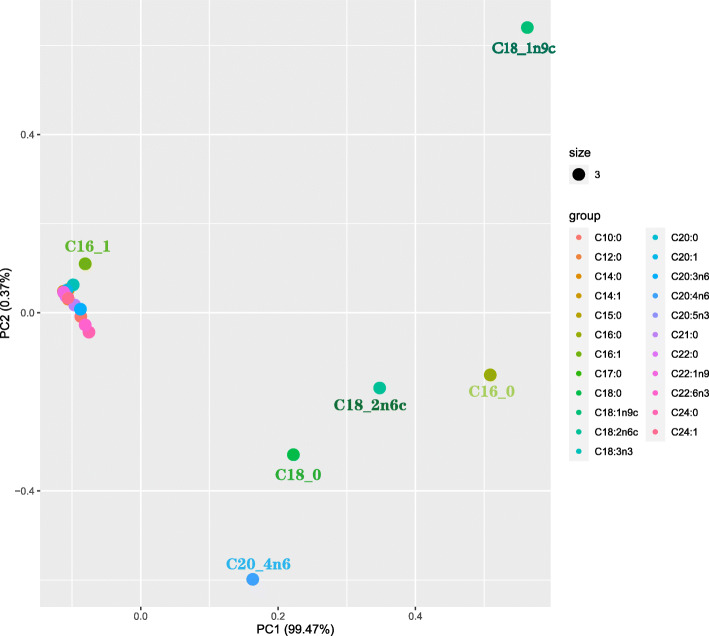
Principal components analysis (PCA) of fatty acid (FA) in chicken meat between the selected line and the control line. PCA based on data of FA composition of meat from all 520 chickens. Data are expressed as the mean ± standard error of mean (SEM); Selected line: *n* = 256; Control line: *n* = 264

**Fig. 4:**
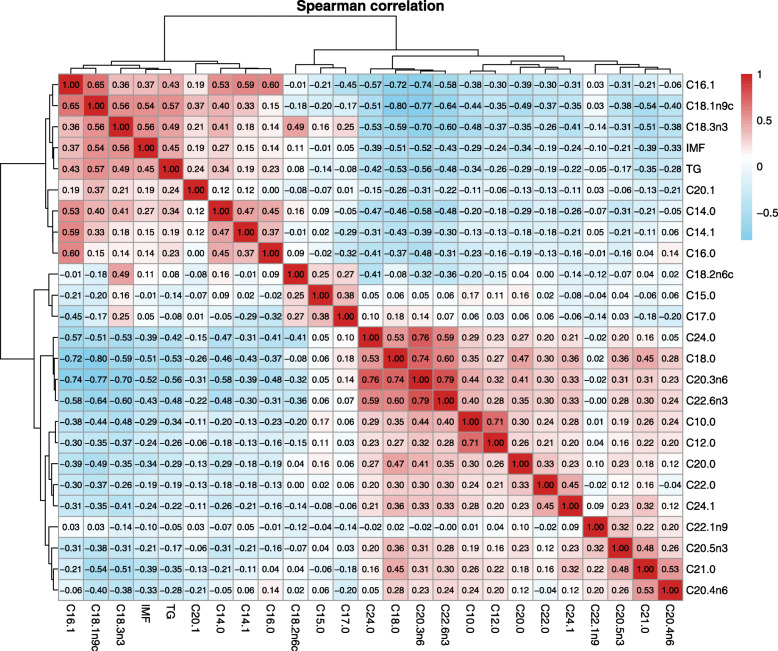
Relationships between IMF, TG, PLIP and fatty acids (FAs). The Spearman correlation analysis was conducted in the R statistics software (version 3.6.1). *n* = 520

A total of 23 common FAs in breast muscle tissue were commonly shared by both the selected line and control line, and the C16:0, C18:0, C18:1n9c, C18:2n6c and C20:4n6 are considered as the main structural components with a proportion exceeding 10% (Table 1). Additionally, the results of the PCA showed that C16:0, C16:1, C18:0, C18:1n9c, C18:2n6c and C20:4n6 were represented in the first principal component, as shown in Fig. 3, suggesting that these long-chain FAs are dominant in the process of IMF deposition. Among 23 common FAs, the proportions of C14:0, C16:1, C18:1n9c and C18:3n3 were significantly increased (*P* < 0.05 or *P* < 0.01), but those of C10:0, C18:0, C20:0, C20:3n6, C20:4n6 and C22:6n3 were significantly decreased (*P* < 0.05 or *P* < 0.01) in the selected line compared to those in the control line (Table 1). In addition, the correlation analysis found positive correlations between TG and IMF and important long-chain FAs (LCFAs, mainly including C14:0, C14:1, C16:0, C16:1, C18:1n9c and C18:3n3), and negative correlations between TG/IMF and other FAs (mainly including C18:0, C20:0, C21:0, C22:0, C20:3n6, C20:4n6, C20:5n3, C22:6n3, C24:0 and C24:1) (Fig. 4 and Additional file 2: Table S2).

**Table 1:** Comparison of fatty acid composition in muscle tissue of the selected line and control line of JXY chicken

Fatty acid, %	Selected line	Control line
C10:0	0.99 ± 0.24^b^	1.05 ± 0.28^a^
C12:0	0.31 ± 0.11	0.32 ± 0.10
C14:0	0.34 ± 0.05^a^	0.31 ± 0.05^b^
C14:1	0.04 ± 0.01	0.04 ± 0.01
C15:0	0.06 ± 0.01	0.06 ± 0.01
C16:0	23.84 ± 0.61	23.75 ± 0.62
C16:1	1.30 ± 0.25^a^	1.21 ± 0.24^b^
C17:0	0.13 ± 0.02	0.14 ± 0.01
C18:0	12.74 ± 0.61^b^	12.97 ± 0.67^a^
C18:1n9c	26.25 ± 1.99^a^	25.39 ± 2.13^b^
C18:2n6c	17.73 ± 1.11	17.56 ± 1.01
C18:3n3	0.60 ± 0.09^a^	0.58 ± 0.09^b^
C20:0	0.17 ± 0.02^b^	0.17 ± 0.03^a^
C20:1	0.23 ± 0.04	0.23 ± 0.06
C21:0	0.67 ± 0.14	0.69 ± 0.15
C20:3n6	0.98 ± 0.13^b^	1.01 ± 0.14^a^
C20:4n6	10.26 ± 1.36^b^	10.96 ± 1.42^a^
C20:5n3	0.15 ± 0.03	0.16 ± 0.03
C22:0	0.19 ± 0.06	0.19 ± 0.05
C22:1n9	0.05 ± 0.02	0.05 ± 0.03
C24:0	1.40 ± 0.27	1.54 ± 0.27
C22:6n3	1.22 ± 0.24^b^	1.30 ± 0.24^a^
C24:1	0.30 ± 0.10	0.30 ± 0.10

Note: ^a,b^Data in the same row with different lowercase letters on the shoulder represented significant difference (*P* < 0.05). *n* = 520 (Selected line: *n* = 256; Control line: *n* = 264)

### FAs in TG determine the total FAs composition of IMF

The breast muscle tissue of each 8 individuals in the groups with high- or low-IMF content were also used to separately extract TG and PLIP to determine the FA composition of IMF. As shown in Table 2, the proportions of C14:0, C16:0, C16:1, C18:1n9c, C18:3n3, and C20:1 were significantly higher, while the proportions of C18:0, C20:0, C20:4n6, C20:4n6, C20:5n3, C22:0, C21:1n9, and C24:0 were significantly (*P* < 0.05 or *P* < 0.01) lower in TGs of the high-IMF group than those of the low-IMF group, which was consistent with the changes of C14:0, C16:0, C16:1, C18:1n9c, C18:3n3, and C20:1 proportions in breast muscle tissue between the high-IMF and low-IMF group. However, only the proportions of C12:0 and C15:0 were significantly different (*P* < 0.05), and the proportions of the remaining FAs did not show significant changes of PLIP in the high-IMF group compared to those in the low-IMF group (*P* > 0.05).

**Table 2:** Fatty acid composition in separately extract IMF, TG and PLIP from muscle tissue of JXY chicken with high- or low-IMF content

Fatty acid, %	IMF		TG		PLIP	
	High IMF	Low IMF	High IMF	Low IMF	High IMF	Low IMF
C12:0	0.278 ± 0.097^b^	0.394 ± 0.145^a^	0.059 ± 0.029	0.060 ± 0.022	0.046 ± 0.007^b^	0.055 ± 0.009^a^
C14:0	0.374 ± 0.066^a^	0.286 ± 0.052^b^	0.314 ± 0.056^a^	0.232 ± 0.028^b^	0.133 ± 0.023	0.148 ± 0.038
C15:0	0.060 ± 0.008	0.067 ± 0.015	0.057 ± 0.009	0.058 ± 0.012	0.086 ± 0.024^b^	0.116 ± 0.025^a^
C16:0	24.128 ± 0.720^a^	23.418 ± 0.591^b^	28.064 ± 1.375	28.262 ± 1.625	25.934 ± 1.300	26.981 ± 1.532
C16:1	1.583 ± 0.189^a^	1.029 ± 0.210 ^b^	1.192 ± 0.229^a^	0.741 ± 0.179^b^	0.249 ± 0.051	0.257 ± 0.054
C17:0	0.135 ± 0.016	0.129 ± 0.015	0.104 ± 0.039	0.106 ± 0.018	0.116 ± 0.02	0.132 ± 0.020
C18:0	11.633 ± 0.438^b^	13.901 ± 0.719^a^	9.546 ± 0.513^b^	11.163 ± 0.713^a^	13.612 ± 0.898	13.866 ± 0.670
C18:1n9c	29.443 ± 1.373^a^	22.831 ± 1.682^b^	26.227 ± 1.979^a^	21.262 ± 1.706^b^	19.290 ± 1.523	18.780 ± 1.345
C18:2n6c	18.009 ± 1.165	17.341 ± 1.309	17.616 ± 1.369	17.316 ± 1.267	13.131 ± 1.319	14.135 ± 1.116
C18:3n3	0.750 ± 0.084^a^	0.471 ± 0.090^b^	0.518 ± 0.132^a^	0.298 ± 0.070^b^	0.049 ± 0.010	0.052 ± 0.015
C20:0	0.147 ± 0.012^b^	0.204 ± 0.028^a^	0.193 ± 0.027^b^	0.233 ± 0.049^a^	0.241 ± 0.031	0.230 ± 0.026
C20:1	0.246 ± 0.018^a^	0.193 ± 0.030^b^	0.182 ± 0.035^a^	0.153 ± 0.017^b^	0.108 ± 0.011	0.102 ± 0.012
C21:0	0.552 ± 0.056^b^	0.752 ± 0.041^a^	0.512 ± 0.044^b^	0.636 ± 0.052^a^	0.586 ± 0.054	0.587 ± 0.071
C20:3n6	0.789 ± 0.093^b^	1.138 ± 0.105^a^	0.998 ± 0.174^b^	1.226 ± 0.113^a^	1.202 ± 0.127	1.185 ± 0.127
C20:4n6	8.206 ± 0.841^b^	12.329 ± 1.363^a^	9.402 ± 1.694^b^	11.959 ± 0.857^a^	18.775 ± 1.519	17.468 ± 1.517
C20:5n3	0.128 ± 0.022^b^	0.177 ± 0.023^a^	0.142 ± 0.028^b^	0.170 ± 0.029^a^	0.299 ± 0.028	0.274 ± 0.046
C22:0	0.178 ± 0.088^b^	0.219 ± 0.041^a^	0.232 ± 0.045	0.273 ± 0.044	0.264 ± 0.058	0.239 ± 0.023
C21:1n9	0.039 ± 0.018^b^	0.073 ± 0.028^a^	1.994 ± 0.319^b^	2.425 ± 1.094^a^	0.078 ± 0.015	0.079 ± 0.014
C24:0	1.098 ± 0.149^b^	1.735 ± 0.249^a^	1.146 ± 0.201^b^	1.540 ± 0.165^a^	2.531 ± 0.357	2.433 ± 0.428
C22:6n3	0.994 ± 0.192^b^	1.510 ± 0.168^a^	1.126 ± 0.248	1.465 ± 0.138	2.875 ± 0.398	2.524 ± 0.394
C24:1	0.288 ± 0.169	0.390 ± 0.176	0.376 ± 0.065	0.421 ± 0.126	0.394 ± 0.066	0.358 ± 0.031

Note: ^a,b^Data in the same row with different lowercase letters on the shoulder represented significant difference (*P* < 0.05). *n* = 16 (Chicken with high-IMF content: *n* = 8; Chicken with low-IMF content: *n* = 8)

### FA synthesis and extracellular intake are jointly involved in IMF deposition

**Fig. 5:**
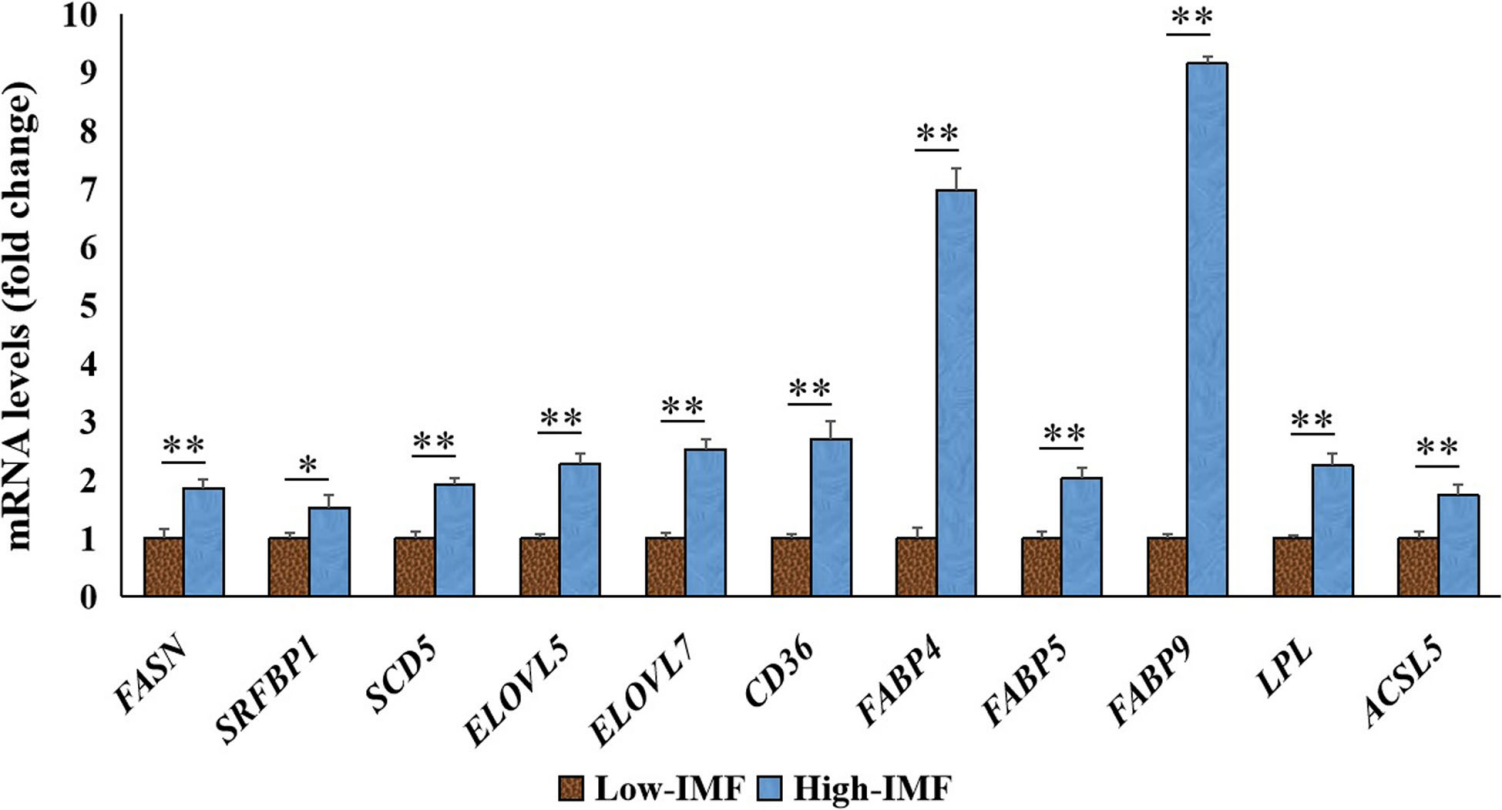
Expression levels of genes related to fatty acid (FA) metabolism in pectoralis of chickens with high- or low-IMF content. Each 8 individuals in the groups with high- or low-IMF content from 520 JXY chickens were used for RNA-sequencing. Genes involved in various processes of FA metabolism, including de novo synthesis (*FASN*, *SRFBP1*), release (*LPL*), desaturation (*SCD*5), elongation (*ELOVL5*, *ELOVL7*), transport (*FABP4*, *FABP5*, *FABP9*, *CD36*) and activation (*ACSL5*). Data are expressed as the mean ± standard error of mean (SEM); *n* = 8

As shown in Fig. 4 and Additional file 2: Table S2, the results of the correlation analysis revealed the higher positive correlation between C14:0 (the important intermediates in de novo synthesis of FA) and C14:1 (*P* < 0.01), C16:0 (*P* < 0.01), C16:1 (*P* < 0.01), C18:1n9c (*P* < 0.01) and C18:3n3 (*P* < 0.01). Also, using our previous RNA-seq data of breast muscle tissue from each 8 individuals in the groups with high- or low-IMF content, some DEGs related to FA synthesis were screened by the following criteria: |log_2_ FC| ≥ 0.58, with *Padj* < 0.05. These DEGs are mainly involved in multiple processes of FA metabolism, including the processes of *DNL* (*FASN*, *SRFBP1*), release (*LPL*), desaturation (*SCD*5), elongation (*ELOVL5*, *ELOVL7*), transport (*FABP4*, *FABP5*, *FABP9*, *CD36*) and activation (*ACSL5*) of FAs. Further, the expression levels of all these genes were significantly (*P* < 0.01) up-regulated in the high-IMF content group compared to those in the low-IMF content group (Fig. 5), and the expression levels of these genes had the significant correlation with the content of IM, TG or FA (Additional file 3: Table S3).

## Discussion

Chicken is the second most consumed meat worldwide by humans, and the quality of meat is also the important demand of people. In the last few years, IMF has received increasing attention due to its major effect on meat quality [26–28], with Kobe beef as a popular example of high-quality grade meat with a high BMS. Our previous studies also demonstrated that the increase of IMF improves the meat quality in JXY chickens [25, 29]. However, the metabolic pathway of chicken IMF deposition is still unclear due to the limitation of the physiological characteristics of chickens. A selected line of JXY chickens has been bred to contain a high IMF content in breast muscle tissue [15], which is critical to the success of scientific research as the suitable experimental model [30]. As expected, we had explained the biochemical mechanism of IMF deposition in chicken in this study by using the populations of the selected line at generation 16 and control line.

A significant increase of the IMF content was confirmed in breast muscle tissue of the selected line at generation 16 compared with that in the control line. Also, the increased IMF was found to promote tenderness and flavor quality in chicken meat as previously reported [15], which confirmed the validity of this selected line model. IMF is a lipid mixture that accumulates in chicken muscle tissue, consisting mainly of TG, PLIP and TCHO [31]. The change of TG content in the two lines (the selected line and the control line) was consistent with the IMF content difference, but not those of PLIP and TCHO, suggesting that the TG content has a more decisive effect on IMF deposition in chicken meat. Also, this view was supported by the correlation analysis for a significant higher positive correlation coefficient between TG content and IMF content (r = 0.45). Thus, we inferred that the TG content was decisive to IMF deposition, although TG and PLIP might together affect the IMF content.

Also, we explored the location of TG and PLIP in the different cell-types, as the main components of IMF. Different from adipocytes as the dominant cell type in abdominal fat tissue, myocytes had an absolute advantage in muscle tissue. Due to the limitations of the method for IMF separation, all lipids in the cells are extracted. PLIP is an essential component of cell membrane [32], so the obtained IMF actually contains the deposited TG in cytoplasm and the structural PLIP from the cytomembrane [17, 18]. The results of in vitro experiments in this study showed that TGs were mainly derived from adipocytes and myocytes, and a certain amount of PLIP were derived from myocytes in muscle tissue.

FA is a structural component of TG and PLIP, and also an important precursor for the flavor volatile compounds [33]. Therefore, we conducted an in-depth investigation of the effect of FAs on the deposition of TG, PLIP, and IMF in the selected line and control line. The results on the FA composition revealed that the long chain FAs (LCFAs), including C16:0, C18:0, C18:1n9c, C18:2n6c and C20:4n6, were more prevalent prevailing with a proportion exceeding 10% in chicken meat as previously reported [34], which can explain the 95.34% phenotypic contribution determined by PCA analysis in this study. Further, by separately isolating TG and PLIP using each eight individuals in the groups with the high- or low-IMF contents, we found that the FA composition of IMF is mainly determined by TG, not by PLIP.

It was known that LCFAs (such as C16:0, C18:0, C18:1n9c, C18:2n6c, et al.) were the main FA composition of fat [34]. In this study, the proportions of LCFAs (C14:0, C16:1, C18:1n9c, C18:3n3, et al.) in the selected line than those of the control line were increased, accompanied with the high positive correlations of TG and IMF. However, the proportions of medium chain FAs (MCFAs) proportions LCFAs (C10:0, C12:0) and very long chain FAs (VLCFAs, C20:0, C21:0, C22:0, C20:3n6, C20:4n6, C20:5n3, C22:6n3, C24:0, C24:1) were decreased in the selected line than those of the control line were found, accompanied with the high negative correlations of TG and IMF. These results suggested that prevalent accumulation of LCFAs and inhibition of MCFAs and VLCFAs jointly resulted in the increase of TG/IMF in the selected line.

The sources of FAs in animal tissues include cellular uptake and DNL. DNL mainly occurs in the liver in poultry [13], and the necessary FAs are mainly provided by cellular uptake in other tissues. In this study, we investigated anew the source of FAs in chicken muscle tissues. Using the above phenotypic correlation analysis based on the FA data of all 520 chickens, the high positive correlation between any two FAs of C14:0, C14:1, C16:0, C16:1, C18:1n9c and C18:3n3 suggested that DNL should occurs in chicken meat. As an auxiliary means, the RNA-seq data of breast muscle tissue from each 8 individuals in the groups with high- or low-IMF content were used, and the representative *FASN* and *SRFBP1* genes related to DNL [35, 36] were screened with the significant higher expression level in the high-IMF group than the low-IMF group. Further, the significant positive correlation between expression levels of two genes (*FASN* and *SRFBP1*) and TG or FAs supported that DNL had contributed to the increase of TG and IMF in chicken muscle tissue.

Also, the expression levels of the representative gene *LPL* related to releasing FAs from blood lipid [10, 11] and *ACSL5* specifically activating FAs from food sources [37] were up-regulated in the high-IMF group, thus insuring the availability of FAs for the cellular uptake option in chicken muscle tissue. In addition, other representative genes (*ELOVL5*, *ELOVL7*, *FABP4*, *FABP5*, *FABP9*, *SCD*5, etc.) related to FAs metabolism were also screened. Among them, FABPs would transport fatty acids to tissues for use or storage [38], and SCD and ELOVLs have an important role in the desaturation or elongation of FAs [39]. Comprehensively considering that the expressions of these genes were significantly up-regulated in the high-IMF group than the low-IMF group, and the significant correlation between gene expression levels and the content of IMF, TG or FA, these results confirmed that FA synthesis was responsible for the increase of TG content in muscle tissue of chickens.

**Fig. 6:**
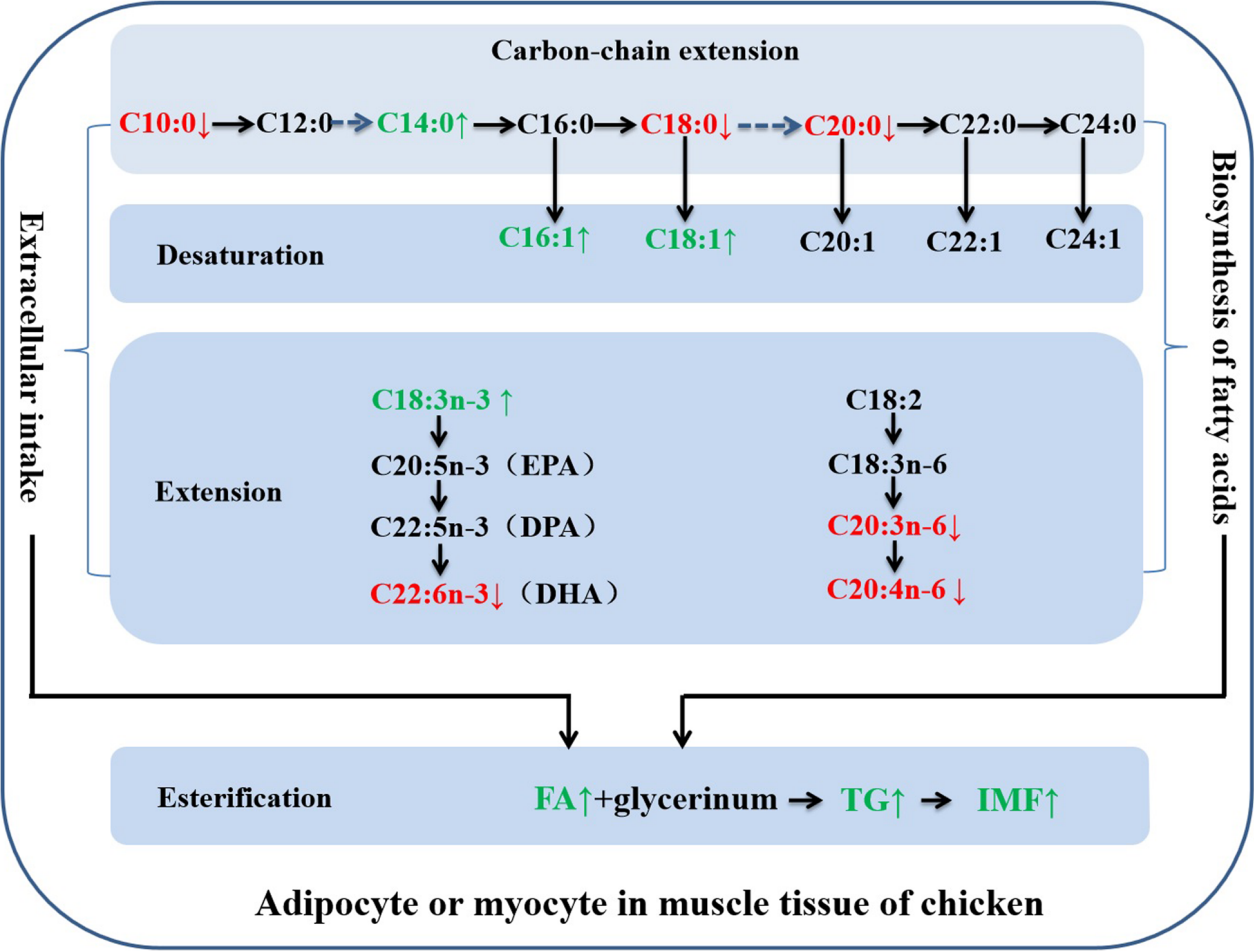
Proposed biochemical mechanism of IMF deposition in chicken meat. This pathway is involved in the biosynthesis of fatty acids (FAs) and triglyceride (TG) in turn. Prevalent accumulation of LCFAs (C16:0, C16:1, C18:1n9c, and C18:2n6c), and inhibitions of MCFAs and VLCFAs would jointly result in the increase of TGs with the FA biosynthesis and cellular uptake ways, and then accelerate the deposition of IMF in chicken meat

In combination with the above results, we mapped out the biochemical metabolic pathway of IMF deposition in chicken, as shown in Fig. 6.

## Conclusions

In summary, our study elucidated the underlying biochemical mechanism of IMF deposition in chicken. Prevalent accumulation of LCFAs (C16:0, C16:1, C18:1n9c, and C18:2n6c), and inhibitions of MCFAs and VLCFAs would jointly result in the increase of TGs with the DNL of FA and cellular uptake ways, and then accelerate the deposition of IMF in chicken meat. Our findings will contribute to improve our understanding of IMF occurrence in chicken, and will provide guidance for the production of high-quality chicken meat.

## Supplementary Information

**Additional file 1: Table S1.** *P*-value of correlation coefficient between IMF and its main components.

**Additional file 2: Table S2.** *P*-value of correlation coefficient between IMF/TG and fatty acids.

**Additional file 3: Table S3.** The correlation analysis between gene expression level and the content IMF, TG and FAs.

## Data Availability

All data generated or analyzed during this study are included in this published article.

## Abbreviations

ACSL5: Acyl-CoA synthetase long chain family member 5
BMS: Beef marbling score
CD36: CD36 molecule
DEGs: Differentially expressed genes
DNL: De novo lipogenesis
ELOVL5: ELOVL fatty acid elongase 5
ELOVL7: ELOVL fatty acid elongase 7
FABP4: Fatty acid binding protein 4
FABP5: Fatty acid binding protein 5
FABP9: Fatty acid binding protein 9
FA: Fatty acid
FAs: Fatty acids
FASN: Fatty acid synthase
GC: Gas chromatography
IMF: Intramuscular fat
JXY: Jingxing Yellow-feather chickens
LCFAs: Long-chain fatty acids
LPL: Lipoprotein lipase
MCFAs: Medium chain fatty acids
PCA: Principal component analysis
PLIP: Phospholipid
SCD5: Stearoyl-CoA desaturase 5
SEM: Standard error of mean
SRFBP1: Serum response factor binding protein 1
TCHO: Total cholesterols
TG: Triglyceride
VLCFAs: Very long chain fatty acids
